# Retraction note: Metastatic ovarian papillary cystadenocarcinoma to the small intestine serous surface: report of a case of high-grade histopathologic malignancy

**DOI:** 10.1186/s13048-016-0286-z

**Published:** 2016-11-04

**Authors:** Fariba Khaki, Javad Javanbakht, Samieh Sharifzad, Mohammad Javad Gharagozlou, Farshid Khadivar, Javad Yaghoobi Yeganeh Manesh, Seyed Hojjat Hosseini, Ali Anissian, Seyed Rashid Touni, Alireza Gilvari, Fatemeh Soghra Abdi

**Affiliations:** 1 Department of Pathology, Faculty of Veterinary Medicine, Tehran University, Tehran, Iran; 2 Department of Clinical Science, Faculty of Veterinary Medicine, Tehran University, Tehran, Iran; 3 Graduate, Faculty of Veterinary Medicine, Tehran University, Tehran, Iran; 4 Gradute of Islamic Azad University of Shahrekord, Faculty of Veterinary Medicine, Shahrekord University, Shahrekord, Iran; 5 Faculty of Veterinary Medicine, Graduate student of Islamic Azad University of Garmsar, Garmsar, Iran; 6 Department of Veterinary, Collage of Agriculture, Abhar Branch, Islamic Azad University, Abhar, Iran; 7 Student of Anatomy and Embryology, Faculty of Veterinary Medicine, Urmia University, Urmia, Iran; 8 Student of Veterinary Medicine, Faculty of Veterinary Medicine, Tehran University, Tehran, Iran; 9 Science and Research Branch of Tehran, Small Animal Internal Medicine Resident of Islamic Azad University, Tehran, Iran

## Retraction

The co-Editors-in-Chief and Publisher have retracted this article [1] because the scientific integrity of the content cannot be guaranteed. An investigation by the Publisher found it to be one of a group of articles we have identified as showing evidence suggestive of attempts to subvert the peer review and publication system to inappropriately obtain or allocate authorship. This article showed evidence of plagiarism (most notably from the articles cited [2–6]) and peer review and authorship manipulation.
